# Phenotype, Biomass, Carbon and Nitrogen Assimilation, and Antioxidant Response of Rapeseed under Salt Stress

**DOI:** 10.3390/plants13111488

**Published:** 2024-05-28

**Authors:** Long Wang, Guobing Lin, Yiyang Li, Wenting Qu, Yan Wang, Yaowei Lin, Yihang Huang, Jing Li, Chen Qian, Guang Yang, Qingsong Zuo

**Affiliations:** 1 Jiangsu Key Laboratory of Crop Genetics and Physiology, Yangzhou University, Yangzhou 225009, China; dx120210110@yzu.edu.cn (L.W.); mx120220714@stu.yzu.edu.cn (G.L.); mx120230775@stu.yzu.edu.cn (Y.L.); 201703106@stu.yzu.edu.cn (W.Q.); 201702420@stu.yzu.edu.cn (Y.W.); 201703107@stu.yzu.edu.cn (Y.L.); hyh226200@163.com (Y.H.); mx120210720@yzu.edu.cn (J.L.); mz120211279@yzu.edu.cn (C.Q.); yangguang@yzu.edu.cn (G.Y.); 2 Jiangsu Co-Innovation Center for Modern Production Technology of Grain Crops, Yangzhou University, Yangzhou 225009, China

**Keywords:** salt stress, phenotype, carbon assimilation, nitrogen assimilation, physiological process, rapeseed

## Abstract

Salt stress is one of the major adverse factors affecting plant growth and crop production. Rapeseed is an important oil crop, providing high-quality edible oil for human consumption. This experiment was conducted to investigate the effects of salt stress on the phenotypic traits and physiological processes of rapeseed. The soil salinity was manipulated by setting three different levels: 0 g NaCl kg^−1^ soil (referred to as S0), 1.5 g NaCl kg^−1^ soil (referred to as S1), and 3.0 g NaCl kg^−1^ soil (referred to as S2). In general, the results indicated that the plant height, leaf area, and root neck diameter decreased with an increase in soil salinity. In addition, the biomass of various organs at all growth stages decreased as soil salinity increased from S0 to S2. The increasing soil salinity improved the distribution of biomass in the root and leaf at the seedling and flowering stages, indicating that rapeseed plants subjected to salt stress during the vegetative stage are capable of adapting their growth pattern to sustain their capacity for nutrient and water uptake, as well as leaf photosynthesis. However, as the soil salinity increased, there was a decrease in the distribution of biomass in the pod and seed at the maturity stage, while an increase was observed in the root and stem, suggesting that salt stress inhibited carbohydrate transport into reproductive organs. Moreover, the C and N accumulation at the flowering and maturity stages exhibited a reduction in direct correlation with the increase in soil salinity. High soil salinity resulted in a reduction in the C/N, indicating that salt stress exerted a greater adverse effect on C assimilation compared to N assimilation, leading to an increase in seed protein content and a decrease in oil content. Furthermore, as soil salinity increased from S0 to S2, the activity of superoxide dismutase (SOD) and catalase (CAT) and the content of soluble protein and sugar increased by 58.39%, 33.38%, 15.57%, and 13.88% at the seedling stage, and 38.69%, 22.85%, 12.04%, and 8.26% at the flowering stage, respectively. In summary, this study revealed that salt stress inhibited C and N assimilation, leading to a suppressed phenotype and biomass accumulation. The imbalanced C and N assimilation under salt stress contributed to the alterations in the seed oil and protein content. Rapeseed had a certain degree of salt tolerance by improving antioxidants and osmolytes.

## 1. Introduction

Rapeseed (*Brassica napus* L.), the second-largest oil crop after soybean, provides edible oil, biodiesel, and other industrial chemicals [1]. The oil derived from rapeseed can produce biodiesel using the alkaline transesterification method [2]. Moreover, the seed contains about 25% protein that can be utilized as animal feed [3]. Hence, rapeseed is a crop with high economic importance. It is crucial to promote the robust and sustainable growth of rapeseed production for the worldwide market of edible oil.

Soil salinization has emerged as a significant problem resulting from both global climate change and human activities, adversely affecting plant growth and crop production [4]. The extent of agricultural land area affected by salinization is progressively expanding on an annual basis. It was reported that 20% of cultivated land and 33% of irrigated land had been salinized [5]. The total saline land area worldwide is increasing at a rate of 10% annually and is projected to account for 50% of arable land by the year 2050 [6]. Therefore, soil salinization poses a great challenge in preventing the sustainable development of agriculture, particularly against the background of rapid urbanization and industrialization.

Plants exposed to salt stress absorb large amounts of salt ion in their tissues, such as sodium (Na^+^) and chloride (Cl^−^) [7,8]. Na^+^ is the predominant cation in saline soil. The previous study reported that, as salinity increased from 0 to 300 mM NaCl L^−1^, leaf Na^+^ content increased, resulting in a decrease in shoot dry weight and plant height in rapeseed [9]. Cl^−^ is a frequently occurring negatively charged ion in saline soil [10]. The high concentration of Cl^−^ may pose negative effects on leaf photosynthesis, resulting in leaf chlorosis [11]. In addition, plants affected by salt stress would overly produce reactive oxygen species (ROS), including peroxide (H_2_O_2_) and superoxide anions (O_2_^−1^) [12]. The considerable accumulation of ROS could lead to oxidative stress, causing cell membrane lipid peroxidation. Salt-tolerant plants employ self-protective mechanisms to mitigate the damage from salt stress [13,14]. For instance, the production of various antioxidants could be improved to promote the ability to eliminate ROS, such as catalase (CAT) and superoxide dismutase (SOD). Generally, SOD acts as a catalyst to reduce O_2_^−1^ to H_2_O_2_, and CAT furtherly reduces H_2_O_2_ to H_2_O [15]. Zhao et al. demonstrated that the activity of POD and SOD in rapeseed significantly increased under 0.6% NaCl conditions compared to the control [16]. However, Zeng et al. found that treatment of 0.75% NaCl marginally increased the leaf POD activity in rapeseed seedlings compared to salt-free conditions, but the difference was not statistically significant [17]. Furthermore, the high salt content in soil could hamper plants from absorbing water, resulting in osmotic stress. Typically, the osmotic potential of roots is generally lower than that of the surrounding soil environment due to the presence of organic substances and inorganic ions within the root system. Consequently, a gradient in osmotic potential drives water influx into the interior of the root system. However, the presence of substantial salt ions in saline soil diminishes and potentially reverses this gradient, thereby impeding water absorption by the roots and leading to osmotic stress. Salt-tolerant plants have the ability to improve the synthesis of a series of osmolytes, including soluble protein and soluble sugar, which helps to alleviate the osmotic stress and avoid cell dehydration [18,19]. Currently, there is abundant research on the phenotypic growth of rapeseed under salt stress; yet, the response of physiology to salt stress remains unclear.

Carbon (C) and nitrogen (N) assimilation are two tightly coupled and critical processes in crop growth and production. The adequate and balanced C and N assimilation is essential for the high yield and high quality of the crops [20]. C assimilation includes photosynthetic carbon fixing and translocation from vegetative organs to reproductive organs [21]. N assimilation comprises N uptake and synthesis of amino acids [22]. N assimilation relies on the availability of a C skeleton produced by C metabolism, while C assimilation needs N to synthesize the essential enzymes involved in C metabolism [23,24]. Salt stress can restrict plant growth and affect photosynthesis and C fixation capacity. High concentrations of salt interfere with leaf physiological functions, leading to decreased chlorophyll content and inhibited photosynthesis and thereby reducing the C assimilation [25]. On the other hand, salt stress may also influence plant N metabolism. The presence of salt in the soil can inhibit the capacity of plant roots to absorb nutrients, leading to a reduction in the availability of N in the soil. Additionally, salt stress may affect N transportation and utilization, further restricting the normal operation of N metabolism pathways [26]. These inhibitory effects may affect C- and N-related physiological processes such as carbohydrate synthesis and protein synthesis. Therefore, understanding the effects of salt stress on C and N assimilation is crucial for elucidating the physiological response mechanisms of rapeseed plants under salt stress.

Therefore, a pot experiment was conducted to investigate the effects of salt stress on rapeseed. The main objectives of this study were as follows: (a) assess the effects of salt stress on phenotype; (b) evaluate the impact of salt stress on biomass and C and N assimilation; (c) investigate the influence of salt stress on antioxidants and osmolytes.

## 2. Results

### 2.1. Effects of Salt Stress on Phenotype

The results of observing phenotypic traits of rapeseed under salt stress are presented in Table 1. The ANOVA results indicated that soil salinity significantly affected all phenotypic traits at the seedling, flowering, and maturity stages. Plant height ranged from 12.27 to 15.59 cm at the seedling stage, 67.15 to 84.09 cm at the flowering stage, and 102.22 to 126.44 cm at the maturity stage. As the soil salinity increased from S0 to S2, plant height significantly decreased by 19.66%, 18.77%, and 16.80% at the seedling, flowering, and maturity stages, respectively. Similarly, root neck diameter was also negatively responsive to soil salinity. As the soil salinity increased from S0 to S2, there was a decrease in root neck diameter by 8.14%, 7.13%, and 8.68% at the respective growth stages. Leaf area across different treatments varied from 83 to 178 cm^2^ plant^−1^ at the seedling stage and from 525 to 978 cm^2^ plant^−1^ at the flowering stage. The impact of soil salinity on leaf area was found to be more significant compared to plant height and root neck diameter. The leaf area reduced by 52.90% at the seedling stage and 45.39% at the flowering stage as soil salinity increased from S0 to S2.

### 2.2. Effects of Salt Stress on Biomass Accumulation and Distribution

**Figure 1:**
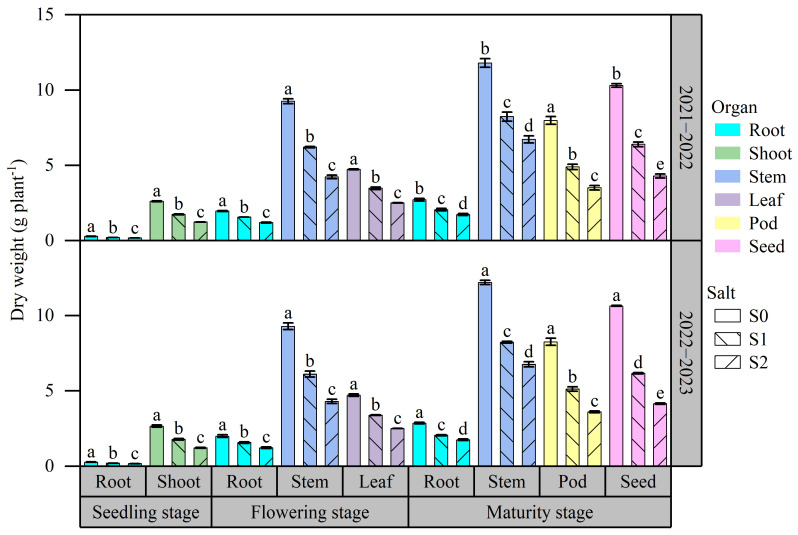
Effects of salt stress on biomass accumulation in various rapeseed organs. Note: Different letters indicate statistically significant differences between different soil salinity levels across two growing seasons (*p* < 0.05). S1: 0 g NaCl kg^−1^ soil; S1: 1.5 g NaCl kg^−1^ soil; S2: 3 g NaCl kg^−1^ soil.

The biomass accumulation of various organs is presented in Figure 1. Under the same soil salinity, the biomass of certain organs during the 2022–2023 growing season was higher than that during the 2021–2022 growing season. The salt stress inhibited biomass accumulation of various organs at all three growth stages. The average biomass of root and shoot at the seedling stage was 0.21 and 1.88 g plant^−1^, respectively. As the soil salinity increased from S0 to S2, there was a significant decrease in the biomass of both the root and shoot by 37.99% and 53.39%, respectively. The biomass of the root, stem, and leaf at the flowering stage ranged from 1.20 to 2.00 g plant^−1^, 4.23 to 9.29 g plant^−1^, and 2.51 to 4.73 g plant^−1^, respectively. The biomass of the root, stem, and leaf in S2 was 38.75%, 54.05%, and 46.88% lower, respectively, than those in S0. The biomass of the root, stem, pod, and seed at the maturity stage ranged from 1.74 to 2.86 g plant^−1^, 6.72 to 12.21 g plant^−1^, 3.51 to 8.27 g plant^−1^, and 4.15 to 10.65 g plant^−1^, respectively. As the soil salinity increased from S0 to S2, there was a considerable decrease in the biomass of different organs during the maturity stage. The root, stem, pod, and seed experienced a drop of 37.32%, 43.86%, 56.21%, and 59.67%, respectively.

The results of biomass distribution in different organs were presented in (Table 2). The ANOVA results showed that soil salinity significantly affected biomass distribution in various organs. At the seedling stage, the biomass distribution in the shoot was significantly higher than that in the root. With an increase in soil salinity from S0 to S2, there was an increase in biomass distribution in the root, reaching a maximum value of 12.18% at S2. In contrast, the biomass distribution in the shoot decreased with increasing soil salinity, reaching a minimum value of 87.81% at S2. At the flowering stage, the majority of the biomass was found in the stem, accounting for 53.28% to 58.05%. The leaf contained the next highest amount of biomass, ranging from 31.24% to 29.65%. The root had the lowest amount of biomass, ranging from 12.38% to 15.27%. As the soil salinity increased from S0 to S2, there was a significant increase in biomass distribution in both root and leaf by 22.58% and 6.32%, respectively. However, there was a drop of 8.04% in biomass distribution in the stem. At the maturity stage, the seed had an average biomass distribution of 38.51%, which was higher than that in the seed (28.83%), pod (23.12%), and root (9.54%). The increasing soil salinity from S0 to S2 significantly decreased biomass distribution in the pod and seed by 10.10% and 17.23%, respectively. However, it increased the biomass distribution in the root and stem by 28.64% and 15.23%, respectively.

### 2.3. Effects of Salt Stress on C and N Assimilation

The results of C accumulation in various organs under different soil salinity levels were presented in (Table 3). Soil salinity significantly affected C accumulation in various organs during both growing seasons. The C accumulation in the root, stem, and leaf at the flowering stage ranged from 0.48 to 0.82 g plant^−1^, 1.60 to 3.68 g plant^−1^, and 0.99 to 1.95 g plant^−1^. At the maturity stage, the C accumulation in the root, stem, pod, and seed ranged from 0.70 to 1.17 g plant^−1^, 2.70 to 5.07 g plant^−1^, 1.41 to 3.38 g plant^−1^, and 2.34 to 6.20 g plant^−1^, respectively. as the soil salinity increased, the C accumulation decreased. Compared to S0, the C accumulation in various organs in S2 exhibited a decrease ranging from 38.52% to 60.92%.

The results of N accumulation in various organs under different soil salinity levels are presented in Table 4. At the flowering stage, the average N accumulation in the root, stem, and leaf was 20.17, 115.34, and 143.18 mg plant^−1^; at the maturity stage, that in the root, stem, pod, and seed was 15.85, 68.75, 43.04, and 258.24 mg plant^−1^, respectively. Soil salinity significantly affected N accumulation in various organs. The order of N accumulation among different soil salinity levels was S0 > S1 > S2. Compared to S0, there was a reduction in N accumulation in various organs by 15.39% to 55.92%.

**Figure 2:**
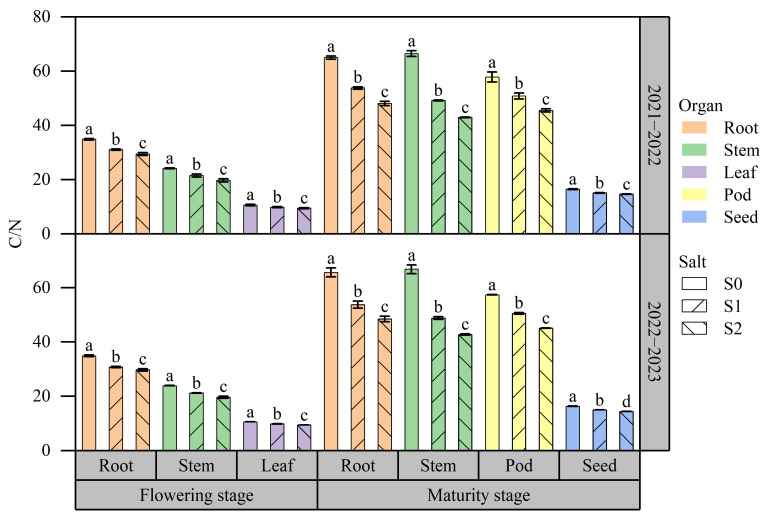
Effects of salt stress on C/N in various organs in rapeseed. Note: Different letters indicate statistically significant differences between different soil salinity values across two growing seasons (*p* < 0.05). S1: 0 g NaCl kg^−1^ soil; S1: 1.5 g NaCl kg^−1^ soil; S2: 3 g NaCl kg^−1^ soil.

Figure 2 shows the results of C/N in various organs across different soil salinity levels. The average C/N across different treatments ranged from 9.44 to 65.70. The C/N exhibited a higher value at the maturity stage compared to the flowering stage. There were differences in C/N among different organs. Specifically, at the flowering stage, the order of C/N in various organs was root > stem > leaf; at the maturity stage, the order was root > stem > pod > seed. Consistent responses to soil salinity were observed in different organs at both growth stages. Higher soil salinity resulted in lower C/N. Compared to S0, the C/N in various organs in S2 decreased by 10.76% to 35.73%.

### 2.4. Effects of Salt Stress on Oil Content and Protein Content

**Figure 3:**
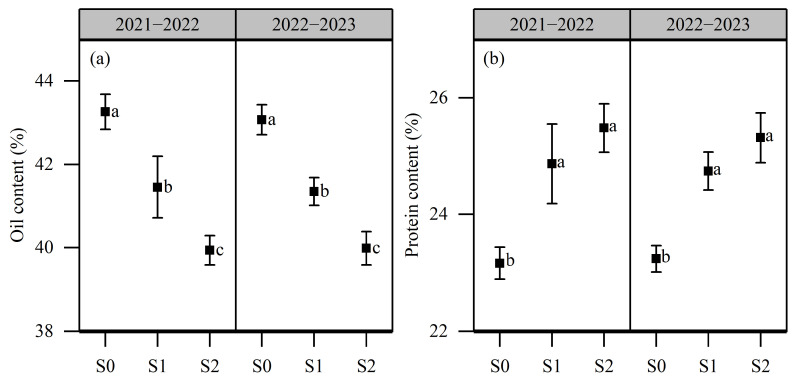
Effects of salt stress on seed oil content and protein content in rapeseed. (**a**): oil content; (**b**): protein content. Note: Different letters indicate statistically significant differences between different soil salinity values across two growing seasons (*p* < 0.05). S1: 0 g NaCl kg^−1^ soil; S1: 1.5 g NaCl kg^−1^ soil; S2: 3 g NaCl kg^−1^ soil.

The results for seed oil content and protein content are presented in Figure 3. The oil content and protein content across different treatments ranged from 39.94% to 43.26% and from 23.16% to 25.48%. There was no significant difference between the two growing seasons. The increase in soil salinity caused a decrease in oil content. The order of oil content across different soil salinity levels was as follows: S0 > S1 > S2. The oil content in S2 decreased by 7.42% relative to S0. Contrastingly, salt stress increased seed protein content. The protein content in S1 was 6.91% higher than in S0. There was no significant difference in protein content between S1 and S2.

### 2.5. Effects of Salt Stress on Antioxidants and Osmolytes

The results of antioxidants and osmolytes at the flowering and maturity stages are presented in Table 5 and Table 6. The activity of SOD and CAT at the seedling stage ranged from 466 to 742 U g^−1^ FW and from 126 to 170 U g^−1^ FW, respectively. Meanwhile, the content of soluble protein and soluble sugar at the seedling stage ranged from 7.55 to 8.77 mg g^−1^ FW and from 103 to 119 mg g^−1^ FW, respectively. At the flowering stage, the SOD activity varied between 479 and 670 U g^−1^ FW, while CAT activity varied between 146 and 187 U g^−1^ FW; the content of soluble protein and soluble sugar was recorded within a range from 8.57 to 9.67 mg g^−1^ FW and 152 to 169 mg g^−1^ FW. The increasing soil salinity level significantly increased the activity of SOD and CAT, as well as the content of soluble protein and soluble sugar, following the order: S2 > S1 > S0.

## 3. Discussion

### 3.1. Response of Phenotypic Traits and Biomass in Rapeseed to Salt Stress

In this study, the phenotypic traits in rapeseed, such as plant height, root neck diameter, and leaf area, significantly decreased with the increasing soil salinity level, which was consistent with the previous report which stated salt stress inhibited plant growth, leading to a decrease in leaf area and dry weight of rapeseed seedlings [27]. These restrained phenotypic traits may result from the disrupted cell elongation and division under salt stress [28]. Root neck diameter is an important index for evaluating the strength of rapeseed plants and is closely related to their ability to withstand lodging. The observed decline in root neck diameter under salt stress indicated that salt stress may increase the risk of lodging. In addition, our study observed a decrease in the biomass of different organs at the seedling, flowering, and maturity stages when soil salinity increased. Our previous studies demonstrated that leaf photosynthesis at the seedling and flowering stage was inhibited under salt stress [29,30], leading to a reduction in biomass accumulation. Our findings differed from those of Bakirv et al., who reported that, compared to the non-NaCl condition, the treatment of 100 mM NaCl L^−1^ showed positive effects on the fresh weight and biomass production in some salt-tolerant varieties [31]. Nevertheless, the majority of research findings support our results, indicating rapeseed phenotypic traits were generally reduced under salt stress conditions, including plant height, dry and fresh weight, and seed yield [32,33].

In this study, it was noted that biomass distribution in different organs varied with the increasing soil salinity. In our study, at the seedling stage, when the soil salinity increased, there was a corresponding increase in the distribution of biomass in the root, while the distribution in the shoot decreased. Our findings align with those of a recent study, which showed that salinity reduced both root and shoot biomass, with a stronger effect on shoot than on root [34]. This may be due to the fact that the decrease in water uptake under salt stress led to less leaf area expansion and transpiration, which in turn maintained the stability of soil moisture to prevent high levels of soil salt [35]. Rapeseed plants can be divided into root, stem, and leaf at the flowering stage. The stem functions to provide a structural support and also acts as a nutrient storage. In comparison, the root and leaf are source organs responsible for nutrient absorption and photosynthesis. Our study found that rapeseed plants exposed to high soil salinity increased the biomass distribution in the root and leaf while decreasing that in the stem at the flowering stage, indicating that rapeseed under salt stress could adjust their growth preferences to improve root and leaf development, thus allowing for the maintenance of nutrient absorption and photosynthetic capacity to a certain extent. Moreover, rapeseed plants under high soil salinity levels at the maturity stage tended to increase the biomass distribution in the root and stem while decreasing that in the pod and seed. These results may be attributed to the restriction of plant growth under salt stress, which affected biomass distribution during their growth. Specially, plants may allocate more resources to support structures such as roots and stems to cope with the survival pressures induced by salt stress. Simultaneously, due to growth limitations, plants may reduce biomass investment in reproductive organs (pod and seed), resulting in decreased distribution proportions. The previous studies aligned with our findings, reporting that salt stress led to a reduction in grain yield and harvest index during the reproductive growth stage [36,37].

### 3.2. Relationship between C and N Assimilation and Seed Quality in Rapeseed under Salt Stress

C and N assimilation are the two important processes affecting plant growth. In this study, the C and N accumulation in various organs at both flowering and maturity stages significantly decreased with increased soil salinity. Zuo et al. found that rapeseed grown in highly saline soil (0.47%) had a 38% drop in C accumulation and a 32.2% decrease in N accumulation compared to rapeseed grown in low-saline soil (0.25%) at the maturity stage [38]. Similar results were observed in other plants, such as rice [39] and soybean [40]. The reductions in C and N accumulation under salt stress may be attributed to several factors. Salt stress decreased C accumulation, presumably by the lower photosynthetic activity caused by ion toxicity and osmotic stress [41]. Moreover, salt stress directly affects the activity of enzymes involved in C and N metabolism, such as Rubisco responsible for C dioxide fixation during photosynthesis as well as nitrate reductase and glutamine synthetase responsible for N metabolism [42,43].

In the present study, the C/N in rapeseed plants decreased as soil salinity increased. Hurtado et al. also reported that the treatment of 100 mM NaCl L^−1^ decreased C/N by 25% in sorghum plants and 17% in sunflower plants compared to the control [44]. Our previous field study also observed that C/N in the high-salt soil decreased compared to low-salt soil, indicating that salt stress disrupted the balance of C and N assimilation [30]. Generally speaking, the C and N assimilation was inhibited by salt stress, more so in C assimilation. In the present pot study, we evaluated the effects of the balance of C and N assimilation on seed quality, including seed oil content and protein content. The coordination between C and N assimilation and regulation of C/N affects carbohydrate and protein synthesis. The previous study demonstrated that seed oil was synthesized through triacylglycerols including several metabolic pathways, such as the Calvin cycle, C assimilation, and starch metabolism [45]. In contrast, seed protein synthesis was associated with N uptake, transportation, and lipid synthesis [46]. The stronger inhibition of salt stress on C assimilation than on N assimilation led to a concentration effect for N, resulting in an increase in N content. Consequently, the seed protein content, associated with the processes of N assimilation, increased, while the seed oil content, linked to the processes of C assimilation, decreased. A similar result was reported wherein rapeseed oil content decreased when salinity was beyond 20 dS m^−1^ [47]. To summarize, salt stress inhibits C and N assimilation, resulting in lower biomass; the seed oil and protein content were altered due to the stronger negative effects of salt stress on C assimilation than N assimilation.

### 3.3. Response of Antioxidants and Osmolytes in Rapeseed to Salt Stress

Previous studies had demonstrated that under salt stress, electrons with high energy would be transferred to molecular O_2_ to cause excessive production of ROS and oxidative stress [9]. In our study, the activity of SOD and CAT at both seedling and flowering stages significantly increased as soil salinity increased. Tian et al. reported that the activity of SOD and CAT significantly increased with an increase in soil salinity from 1.5 to 7.5 g NaCl kg^−1^ soil [48]. Similarly, Yildiz et al. also recorded a significant increase in the activity of SOD and CAT under the treatment of 150 mM NaCl L^−1^ compared to the control [49]. However, some studies indicated that salt stress had no effect or negative effects on antioxidants. The possible reason for this discrepancy may lie in the fact that different varieties possess varying degrees of salt tolerance. For instance, Kumar et al. reported that as NaCl concentration increased from 0 to 150 mM NaCl L^−1^, for the salt-tolerant variety of rapeseed, the activity of SOD and CAT significantly increased, while for the salt-sensitive variety, the SOD activity decreased, and the CAT activity showed no significant difference [50]. Therefore, the cultivation and selection of salt-tolerant varieties are vital for rapeseed production in saline soil.

The presence of salt ions in saline soil can decrease the osmotic potential of the soil and thus inhibit water uptake by the plant roots. The accumulation of soluble substances is an effective way to alleviate osmotic stress and improve salt tolerance. Soluble sugar and soluble protein are the most significant osmolytes actively participating in osmoregulation under stress conditions. In our study, up-regulation of the content of soluble protein and soluble sugar was recorded as the soil salinity level increased. Similar results were reported by EI-Badri et al. who observed that the content of soluble sugar and soluble protein in rapeseed seedlings under saline conditions significantly increased by 40.68–43.96% and 88.89–89.04% compared to the control [51]. The accumulation of these osmolytes can maintain the cell osmotic potential to adjust to the saline environment. In addition, the antioxidant (except SOD) and osmolyte levels at the flowering stage were greater than at the seedling stage, indicating that the salt tolerance of rapeseed plants enhanced as the growth progressed. Therefore, rapeseed exposed to a certain range of soil salinity could increase the antioxidants and osmolyte levels to counteract salt stress.

## 4. Materials and Methods

### 4.1. Experiment Design

This pot experiment was conducted at Yangzhou University Experimental Farm, Yangzhou, Jiangsu province, China, during the 2021–2022 and 2022–2023 growing seasons. The variety Qinyou10 was used in this experiment. The size of the pot used in the experiment was 35 cm × 30 cm (height × diameter); these pots were without holes in the bottom to avoid the leakage of nutrients and salt. Each pot contained 10 kg of soil. The soil was derived from the plow layer with pH 7.1, organ matter 0.134 g kg^−1^, total N 1.2 g kg^−1^, available P 13.8 mg kg^−1^, and available K 80.1 mg kg^−1^.

This experiment was arranged with a completely randomized design with three levels of soil salinity, with three replicates (each replicate had 10 pots). The soil salinity was developed through adding sodium chloride (NaCl) into soils, including 1.5 g NaCl kg^−1^ soil (S1) and 3.0 g NaCl kg^−1^ soil (S2). The soil without adding NaCl was non-salt stress (S0). The relative quantity of NaCl was dissolved in water, which was added into soil before sowing. Then, the soil was mixed fully to ensure the saline was well-distributed in the pot. A total of 10 seeds per pot were sown on 15 October each year. The density was adjusted to 3 plants per pot at the third and fourth leaf stages. The urea, triple-superphosphate, and potassium sulfate were applied at a rate of 0.20, 0.20, and 0.24 g kg^−1^ soil as basal fertilizer. The urea was applied at a rate of 0.20 g kg^−1^ soil as bolting fertilizer. All pots were placed in the awning. To prevent rainwater infiltration into the pots, a plastic film was deployed above the awning when rainfall was anticipated, and promptly removed once the rain ceased. Tap water served as the irrigation source throughout the experiment.

### 4.2. Sample and Measurement

#### 4.2.1. Phenotypic Traits and Biomass

The sample was collected at the seedling, flowering, and maturity stage. For each treatment at each growth stage, a total of 9 plants were collected from 3 pots. Plant height, leaf area, and root neck diameter were measured. Then, the plants were separated into root and shoot at the seedling stage, root, stem, and leaf at the flowering stage, and root, stem, pod, and seed at the maturity stage. Afterward, the samples were dried at 105 °C for 30 min, then at 85 °C until a consistent weight for biomass measurement was reached. The biomass distribution in a specific organ was calculated by dividing the dry weight of this organ by the total dry weight of the whole plant.

#### 4.2.2. Content and Accumulation of C and N

The above samples were used to determine the C and N content. The same organ from three plants in the same pot was mixed and pulverized. Then, the C and N content of specific organs at the flowering and maturity stages were measured by the elemental analyzer (Vario MAX CN, Elementar Co., Langenselbold, Germany). The C and N accumulation was calculated by multiplying the C and N content by the dry weight of the specific organ. The C/N was calculated by dividing the C content by the N content of the specific organ.

#### 4.2.3. Antioxidants and Osmolytes

The top second and third fully expanded leaves at the seedling and flowering stages were sampled under optimal weather conditions and promptly frozen using liquid N and stored in a low-temperature freezer for the antioxidant and osmolyte measurements, including SOD, CAT, soluble protein, and soluble sugar. Each trait was measured three times. These physiological traits were measured using the enzyme-linked immunosorbent assay (ELISA) provided by Shanghai Enzyme-linked Industrial Co., Ltd., Shanghai, China. The fresh leaf sample (1 g) was mixed with 9 mL of a 50 mM/L phosphate buffer solution (pH = 7.8). Simultaneously, the mixture was combined with quartz sand, ground under ice conditions, and then centrifuged at 15,000× *g* r/min for 20 min at 4 °C. The supernatant was prepared and mixed with the standard substrate, followed by a reaction for 30 min at 37 °C. Subsequently, the plate underwent five washes before adding and reacting with the enzyme reagent for another 30 min at 37 °C; after this step, the plate was rinsed five times again. Next, a stain was added and reacted for 10 min at 37 °C, followed by the addition of the stop solution. Finally, the optical density (OD) value was measured at a wavelength of 450 nm.

### 4.3. Statistical Analysis

For phenotypic traits, the average value of three measurements taken from three plants in the same pot was considered as one repetition; then, three average values from three pots were used for statistical analysis. For other traits, three measured values were used for analysis. The data were compiled with Microsoft Excel 365 (Microsoft Corp., Redmond, WA, USA) and analyzed using R-4.3.1. Analysis of variance (ANOVA) was performed, and the means were compared by the least significant difference (LSD) at *p* = 0.05 using the Agricolae package. The graphs were performed using Originpro2024 software (OriginLab Corp., Northampton, MA, USA).

## 5. Conclusions

Salt stress affected growth and physiological traits in rapeseed. In general, plant height, leaf area, and root neck diameter showed a significant reduction as soil salinity increased. In addition, the increasing soil salinity decreased biomass accumulation by inhibiting C and N assimilation. In addition, at the seedling and flowering stages, the biomass distribution in the root and leaf under high soil salinity levels was raised; however, at the maturity stage, biomass distribution in the root and stem increased while that in the pod and seed decreased with the increasing soil salinity. Moreover, the imbalanced C and N assimilation due to salt stress led to reduced oil content and increased protein content. Furthermore, rapeseed plants exposed to salt stress could stimulate antioxidants and osmolytes to eliminate ROS and maintain cell water potential. Therefore, salt stress decreased biomass accumulation and changed seed oil and protein content by interfering with C and N assimilation; rapeseed plants showed tolerance by promoting antioxidants and osmolytes.

## Figures and Tables

**Table 1:** Effects of salt stress on phenotypic traits in rapeseed.

Year	Soil Salinity	Seedling Stage			Flowering Stage			Maturity Stage	
		Plant Height (cm)	Root Neck Diameter (mm)	Leaf Area (cm^2^ Plant^−1^)	Plant Height (cm)	Root Neck Diameter (mm)	Leaf Area (cm^2^ Plant^−1^)	Plant Height (cm)	Root Neck Diameter (mm)
2021–2022	S0	15.14 a	2.72 a	174.0 b	82.46 a	14.63 a	967 a	121.90 b	14.95 a
	S1	13.70 b	2.60 bc	118.6 c	74.08 b	14.00 b	713 b	111.49 c	14.33 b
	S2	12.27 c	2.50 d	82.5 d	67.15 c	13.50 c	525 c	104.28 d	13.53 c
2022–2023	S0	15.59 a	2.76 a	177.9 a	84.09 a	14.46 a	978 a	126.44 a	15.01 a
	S1	13.91 b	2.61 b	121.4 c	73.61 b	13.95 b	718 b	113.08 c	14.23 b
	S2	12.42 c	2.53 cd	83.2 d	68.13 c	13.52 c	536 c	102.22 d	13.82 bc
ANOVA									
Year		*	ns	*	ns	ns	ns	ns	ns
Soil salinity		**	**	**	**	**	**	**	**
Year × soil salinity		ns	ns	ns	ns	ns	ns	*	ns

Note: Different letters indicate statistically significant differences between treatments (*p* < 0.05). ns, not significant; * and ** indicate *p* < 0.05 and 0.01. S1: 0 g NaCl kg^−1^ soil; S1: 1.5 g NaCl kg^−1^ soil; S2: 3 g NaCl kg^−1^ soil.

**Table 2:** Effects of salt stress on biomass distribution (%) in various organs in rapeseed.

Year	Soil Salinity	Seedling Stage		Flowering Stage			Maturity Stage			
		Root	Shoot	Root	Stem	Leaf	Root	Stem	Pod	Seed
2021–2022	S0	9.45 c	90.55 a	12.30 c	58.05 a	29.65 bc	8.27 c	35.97 c	24.31 a	31.45 a
	S1	10.07 b	89.93 b	13.94 b	55.17 b	30.89 a	9.53 b	38.15 b	22.69 b	29.63 b
	S2	12.15 a	87.85 c	15.11 a	53.28 c	31.61 a	10.67 a	41.32 a	21.57 c	26.44 c
2022–2023	S0	9.44 c	90.56 a	12.49 c	58.05 a	29.46 c	8.43 c	35.91 c	24.32 a	31.34 a
	S1	10.08 b	89.92 b	14.17 b	55.21 b	30.62 ab	9.54 b	38.19 b	23.71 a	28.56 b
	S2	12.22 a	87.78 c	15.27 a	53.49 c	31.24 a	10.8 a	41.51 a	22.15 bc	25.53 c
ANOVA										
Year		ns	ns	ns	ns	ns	ns	ns	*	ns
Soil salinity		**	**	**	**	**	**	**	**	**
Year × salt		ns	ns	ns	ns	ns	ns	ns	ns	**

Note: Different letters indicate statistically significant differences between treatments (*p* < 0.05). ns, not significant; * and ** indicate *p* < 0.05 and 0.01. S1: 0 g NaCl kg^−1^ soil; S1: 1.5 g NaCl kg^−1^ soil; S2: 3 g NaCl kg^−1^ soil.

**Table 3:** Effects of salt stress on C accumulation (g plant^−1^) in various organs in rapeseed.

Year	Soil Salinity	Flowering Stage			Maturity Stage			
		Root	Stem	Leaf	Root	Stem	Pod	Seed
2021–2022	S0	0.81 a	3.68 a	1.95 a	1.11 b	4.90 a	3.26 a	6.00 b
	S1	0.64 b	2.39 b	1.40 b	0.83 c	3.34 b	1.98 b	3.64 c
	S2	0.48 c	1.60 c	0.99 c	0.70 d	2.70 c	1.41 c	2.42 e
2022–2023	S0	0.82 a	3.68 a	1.94 a	1.17 a	5.07 a	3.38 a	6.20 a
	S1	0.64 b	2.35 b	1.36 b	0.83 c	3.35 b	2.07 b	3.51 d
	S2	0.49 c	1.62 c	0.99 c	0.71 d	2.72 c	1.44 c	2.34 e
ANOVA								
Year		ns	ns	ns	ns	ns	ns	ns
Soil salinity		**	**	**	**	**	**	**
Year × soil salinity		ns	ns	ns	ns	ns	ns	**

Note: Different letters indicate statistically significant differences between treatments (*p* < 0.05). ns, not significant; ** indicates *p* < 0.01. S1: 0 g NaCl kg^−1^ soil; S1: 1.5 g NaCl kg^−1^ soil; S2: 3 g NaCl kg^−1^ soil.

**Table 4:** Effects of salt stress on N accumulation (mg plant^−1^) in various organs in rapeseed.

Year	Soil Salinity	Flowering Stage			Maturity Stage			
		Root	Stem	Leaf	Root	Stem	Pod	Seed
2021–2022	S0	23.19 a	152.68 a	184.46 a	17.13 a	73.71 ab	56.41 a	365.07 b
	S1	20.52 b	111.44 b	142.34 b	15.49 b	68.01 cd	39.07 b	241.46 c
	S2	16.40 c	81.02 c	105.10 c	14.54 b	62.97 d	30.90 c	165.42 e
2022–2023	S0	23.61 a	153.88 a	183.73 a	17.88 a	75.79 a	58.89 a	380.33 a
	S1	20.72 b	110.66 b	138.62 b	15.51 b	68.54 bc	41.01 b	234.17 d
	S2	16.60 c	82.39 c	104.85 c	14.56 b	63.5 cd	31.96 c	162.97 e
ANOVA								
Year		ns	ns	ns	ns	ns	ns	ns
Soil salinity		**	**	**	**	**	**	**
Year × salt		ns	ns	ns	ns	ns	ns	**

Note: Different letters indicate statistically significant differences between treatments (*p* < 0.05). ns, not significant; ** indicates *p* < 0.01. S1: 0 g NaCl kg^−1^ soil; S1: 1.5 g NaCl kg^−1^ soil; S2: 3 g NaCl kg^−1^ soil.

**Table 5:** Effects of salt stress on antioxidants and osmolytes at the seedling stage in rapeseed.

Year	Soil Salinity	SOD (U g^−1^ FW)	CAT (U g^−1^ FW)	Soluble Protein (mg g^−1^ FW)	Soluble Sugar (mg g^−1^ FW)
2021–2022	S0	467 c	126 c	7.63 c	104 c
	S1	625 b	154 b	8.19 b	114 b
	S2	735 a	170 a	8.77 a	119 a
2022–2023	S0	466 c	126 c	7.55 c	103 c
	S1	621 b	152 b	8.22 b	113 b
	S2	742 a	167 a	8.77 a	118 a
ANOVA					
Year		ns	ns	ns	ns
Soil salinity		**	**	**	**
Year × salt		ns	ns	ns	ns

Note: Different letters indicate statistically significant differences between treatments (*p* < 0.05). ns, not significant; ** indicates *p* < 0.01. S1: 0 g NaCl kg^−1^ soil; S1: 1.5 g NaCl kg^−1^ soil; S2: 3 g NaCl kg^−1^ soil.

**Table 6:** Effects of salt stress on antioxidants and osmolytes at the flowering stage in rapeseed.

Year	Soil Salinity	SOD (U g^−1^ FW)	CAT (U g^−1^ FW)	Soluble Protein (mg g^−1^ FW)	Soluble Sugar (mg g^−1^ FW)
2021–2022	S0	485 c	154 d	8.63 c	157 cd
	S1	591 b	173 bc	9.02 b	159 bc
	S2	670 a	187 a	9.60 a	165 ab
2022–2023	S0	479 c	146 d	8.57 c	152 d
	S1	606 b	166 c	9.07 b	159 bcd
	S2	650 a	181 ab	9.67 a	169 a
ANOVA					
Year		ns	**	ns	ns
Soil salinity		**	**	**	**
Year × salt		*	ns	ns	ns

Note: Different letters indicate statistically significant differences between treatments (*p* < 0.05). ns, not significant; * and ** indicate *p* < 0.05 and 0.01. S1: 0 g NaCl kg^−1^ soil; S1: 1.5 g NaCl kg^−1^ soil; S2: 3 g NaCl kg^−1^ soil.

## Data Availability

Data are contained within the article.
